# Agricultural practices can threaten soil resilience through changing feedback loops

**DOI:** 10.1038/s44264-025-00098-6

**Published:** 2025-10-01

**Authors:** Alison M. Carswell, Simon Willcock, Martin S. A. Blackwell, Hari Ram Upadhayay, Paul Harris, Graham McAuliffe, Andrew L. Neal, M. Jordana Rivero, Laura M. Cardenas, Stephan M. Haefele, Andrew P. Whitmore, John A. Dearing, Fusuo Zhang, Mark Farrell, Marijn Bauters, Pascal Boeckx, Yuri Jacques A. B. da Silva, Kwame Agyei Frimpong, Adrian L. Collins

**Affiliations:** 1 Net Zero & Resilient Farming, Rothamsted Research, North Wyke, Okehampton UK; 2 School of Environmental and Natural Sciences, Bangor University, Bangor, Gwynedd UK; 3 Sustainable Soils and Crops, Rothamsted Research, Harpenden, UK; 4 Net Zero & Resilient Farming, Rothamsted Research, West Common, Harpenden, UK; 5 School of Geography and Environmental Science, University of Southampton, Southampton, UK; 6 State Key Laboratory of Nutrient Use and Management, College of Resources and Environmental Sciences, National Academy of Agriculture Green Development, National Observation and Research Station of Agriculture Green Development (Quzhou, Hebei), China Agricultural University, Beijing, China; 7 CSIRO Agriculture & Food, Kaurna Country, Adelaide, SA Australia; 8 UWA School of Agriculture & Environment, The University of Western Australia, Whadjuk Noongar Country, Perth, WA Australia; 9 Department of environment, Ghent University, Ghent, Belgium; 10 Department of green Chemistry and Technology, Ghent University, Ghent, Belgium; 11 Agricultural Engineering Department, Federal Rural University of Pernambuco, Recife, Brazil; 12 Department of Soil Science, School of Agriculture, College of Agriculture and Natural Sciences, University of Cape Coast, Cape Coast, Ghana

**Keywords:** Environmental impact, Element cycles, Agroecology

## Abstract

Soil has supported terrestrial food production for millennia; however, agricultural intensification may affect its resilience. Using a systems-thinking approach, we reviewed the impacts of conventional-agriculture practices on soil resilience and identified alternative practices that could mitigate these effects. We found that many practices only affect soil resilience with their long-term repeated use. Lastly, we ranked the impacts that pose the greatest threats to soil resilience and, consequently, food and feed security.

## Introduction

### Resilience theory

Resilience theory describes a spectrum of system responses to drivers or perturbations from gradual, near-linear and reversible, to abrupt, non-linear and strongly hysteretic^1^. The position of a system on this spectrum is affected by the nature of feedback loops. Resilient systems are dominated by balancing (or negative) feedback loops that absorb short- and long-term stress without changing the overall system structure and function. As stress increases, the underlying feedback mechanisms may change from net balancing to net reinforcing via a strengthening of existing reinforcing (or positive) feedback loops, emergence of new reinforcing feedback loops, or via a weakening of existing balancing feedback loops. Systems with the strongest net reinforcing feedback mechanisms are associated with the strongest hysteretic effects^2^, meaning that they may be the most difficult to restore. Furthermore, stronger interactions between systems may be expected to increase the number of the ‘cascades of collapse’, wherein loss of resilience in one system triggers a domino effect resulting in degradation across many other systems^3^.

Simplified, more homogenised structures – such as agroecosystems – may have fewer internal balancing feedback loops and a reduced ability to reconfigure quickly in the face of stress; i.e., are potentially low-resilience systems. However, agroecosystems exist under human management. Short-term management interventions tend to maintain or stabilise the system (e.g., irrigation in place of limited rainfall) but may unwittingly erode internal balancing feedback loops (e.g., tilling soil leading to degraded soil structure and soil organic matter (OM) loss) upon which the resilience of agricultural soils to future stresses could ultimately depend^3^. Eroding or removing balancing feedback loops could lead to long-term growth in the influence of reinforcing feedback loops accompanied by vicious circles, characterised by increases in salinity, pests, weeds, diseases and decreases in crop yield or plant species persistency – all made worse when management interventions are inaccessible (e.g., when geopolitical shocks delay access to agrochemicals). Similarly, in the short-term, management practices have the potential to strengthen existing reinforcing feedback loops, or to drive emergence of new reinforcing feedbacks. Thus, recent developments in systems thinking can help provide insight into how best to manage agriculture for resilient soils^4^, ranging from highlighting potential interventions and feedback loops to system interactions, as well as near-irreversible effects (discussed below).

### Resilience of agricultural soils

This work is intended to demonstrate how resilience theory can be applied to the most prevalent global agricultural production systems, through reviewing academic literature and determining the major threats to soil resilience. Not all agricultural production systems could be covered within one article and the absence of practices that impact soil resilience, such as peatland drainage for cultivation and forestry and soil compaction caused by heavy machinery use, is acknowledged. We envisaged that subsequent work could explore in more detail the complexity of different types of systems, management, and the impacts and feedback loops that occur within, or as a result of, them.

We focus on the effects of agricultural practices on the resilience of soils as they, either directly or indirectly, support 95% of food production^5^, are the largest terrestrial store of carbon on the planet (1700 Gt^6^) and provide habitat for as much as 59% of the Earth’s species^7^. Healthy soils provide a variety of functions and services that are directly or indirectly related to soil structure, soil biota and its biodiversity^8,9^. Soil resilience has been defined in earlier research^10,11^. However, human-induced soil degradation, combined with climate change, is compromising soil resilience and undermining our ability to achieve Sustainable Development Goals (SDG) associated with soil, such as zero hunger, clean water and sanitation and life on land. Various threats to soil have been identified globally, including elevated erosion, OM loss, salinization, acidification, contamination, biodiversity loss, nutrient imbalance, compaction, sealing, and loss of moisture^12^. These threats are strongly interrelated and linked through powerful feedback loops and are responsible for pervasive soil degradation^13^. Most of the world’s soil resources are in only fair, poor, or very poor states; over three-quarters of the Earth’s land area is degraded in some way, and one-third is moderately to highly degraded [12, IPBES, 2018^14^). Soil degradation incurs substantial economic loss both in developed and developing countries. The areas of land experiencing losses of ecosystem services associated with land degradation have been estimated to range 16,746 or 29,623 USD km^-2^ for Oceania to 224,434 or 124,191 USD km^-2^ for Asia, according to the Imhoff or Haberl model, respectively, with unsustainable agricultural practices cited as the typical cause (ELD Initiative, 2015^15^). However, there are wider implications of many of the agricultural management practices outside of the soil system; for example, production of greenhouse gases and air pollutants, transfer of nutrients and other pollutants to surface and ground waters, or energy requirements for producing agrochemicals or other agricultural products, which can ultimately feed back into soil processes. Here, our focus was on soil, the processes occurring immediately within it, and the implications of conventional agricultural management practices for soil resilience. We used the capacity for soil to support agricultural productivity as crop or meat and milk yields as our measure of soil resilience. Although we note that wider external feedbacks, such as climate change, will also affect the long-term sustainability of agricultural production.

## Conceptualising the sustainability of farm management practices on soils

**Fig. 1:**
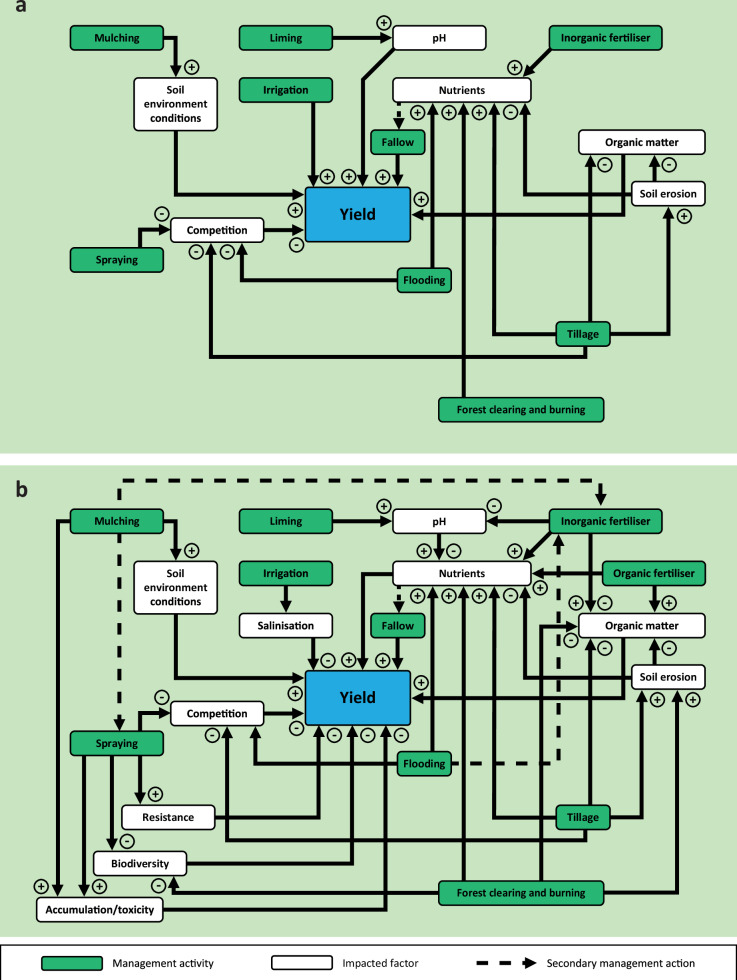
Impacts of agricultural management practices in cropping systems in the short-term. **a** and in the long-term (**b**) and how multiple management tools are used to address the long-term impacts of management activities and maintain crop yields. The actions of the farmer (i.e., leading to the management intervention, often in response to yields or profits) is shown with a dashed arrow, while the knock-on effects of these actions are arrived at by solid black arrows. The positive or negative symbols indicate the direction of the relationship between the two factors (e.g., a ‘+’ showing a positive relationship between two factors, such as liming and pH, and a ‘-‘ indicating a negative relationship, such as between spraying and competition). Grazing systems are excluded due to the divergent nature of livestock systems versus cropping systems (see Fig. 2). See supporting information for links between management activity and impacts.

**Fig. 2:**
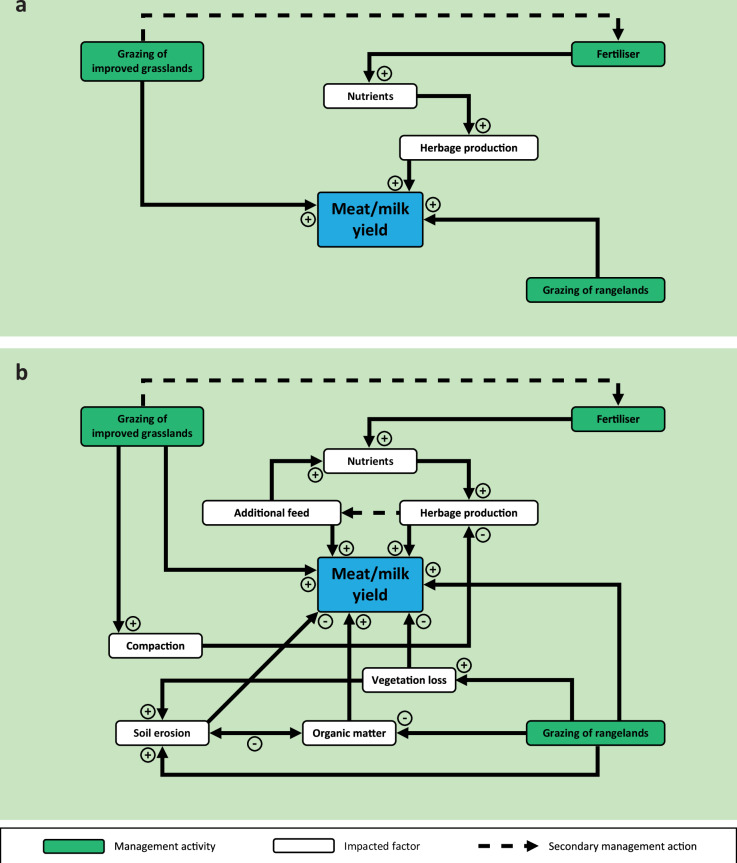
Impacts of agricultural management practices in grazing systems in the short-term. **a** and in the long-term (**b**) and how multiple management tools are used to address the impacts of long-term management practices and maintain meat and/or milk yields. The actions of the farmer (i.e., leading to the management intervention, often in response to yields or profits) is shown with a dashed arrow, while the knock-on effects of these actions are arrived at by solid black arrows. The positive or negative symbols indicate the direction of the relationship between the two factors (e.g., a ‘+’ showing a positive relationship between two factors, such as fertiliser and nutrients, and a ‘-‘ indicating a negative relationship between two factors, such as soil erosion and organic matter). See supporting information for links between management activity and impacts.

To provide insight into the short- and long-term impacts of conventional agricultural management practices on agricultural soils, we constructed system diagrams focusing on effects reported in the scientific literature of the most common and widespread agricultural management practices: tillage; application of nitrogen (N) fertiliser; liming; application of pesticides; plastic mulch films; irrigation; flooding; extensive and intensive grazing and shifting cultivation (Supplementary Figs. 1−10). From these, we considered the potential balancing and reinforcing feedbacks resulting from each management practice across both short- and long-term timeframes, highlighted the direction of reported effects and identified cycles where reinforcing feedback loops may be observed and how they might affect yields (Supplementary Figs. 1−10). The systems diagrams were used to demonstrate the long-term impacts of crop and grazing management systems (Figs. 1 and 2 and described in full in the Supplementary Information, Supplementary Figs. 1−10) and how multiple management interventions are used to maintain crop, meat and milk yields. The short- and long-term impacts of the listed agricultural practices are briefly summarised below. In each case, the starting point is considered to be a soil under natural or semi-natural vegetation, which is converted to agricultural production. We note that the external impacts of the practices examined are many and can also affect soil resilience; however, these were outside the scope of our analysis and have not been included. Instead, this research is focused on the direct effects of conventional agricultural management practices on soil resilience.

### Tillage

Tillage is a common practice that inverts soil to a predetermined depth and is carried out to reduce competition from other plants, to improve conditions for seed development, and to improve access to soil nutrients (see Supplementary Fig. 1 for further details). In the short-term, there are few consequences for yield arising from conventional tillage, and a yield gain can be achieved due to the mineralisation of soil OM into nutrition that is readily available for crop uptake, so the practice continues with no remedial management (Supplementary Fig. 1a). There are negative external impacts associated with tillage in the short-term^16,17^ which include elevated soil erosion, nutrient losses via leaching and gaseous emissions, mortality of beneficial invertebrates (e.g., earthworms) and the disruption of fungal hyphal networks. However, the impacts of these on soil resilience are more likely to become consequential after repeated tillage rather than in the short term.

Over the long-term, repeated tillage leads to declines in soil OM, soil nutrient supply, beneficial invertebrates and fungal hyphal networks, soil aggregate stability and potential development of plough pans^16,18,19^ (which can be addressed using deep tillage) (Supplementary Fig. 1b). These biogeochemical and physical changes to the soil result in a positive reinforcing feedback loop, whereby declines in soil OM leads to declining crop yields and soil erosion, potentially resulting in increased dependence on tillage (potentially deeper tillage) or fertiliser inputs to maintain crop yields (see also Supplementary Fig. 2) or a change in management practices^17,20^ (Table 1).

### Application of fertilisers

Fertilisers can be split broadly into two groups. First, fertilisers are derived from organic residues such as livestock manures, composts, sewage sludge, and other recycled organic by-products. The second fertiliser category is sourced via industrial synthesis or mineral extraction (see Supplementary Information for more detail). We focus on N as the primary macronutrient added to soils globally; however, soil applications of mined nutrients such as phosphorus are also highly relevant for the long-term resilience of soils^21^. The short-term effects of applying synthetic N fertiliser, where it is limiting, are typically beneficial for crop yields^22^ (Supplementary Fig. 2a). The consequence of this positive crop response to N fertilisation is to continue applying it as a routine practice.

Over the long term, continued use of synthetic N fertilisers has a complicated relationship with crop yield (Supplementary Fig. 2b). Vonk et al.^23^ demonstrated that crop N uptake can increase over continuous years of N fertilisation, due to the legacy of increased soil N supply. However, increasing application rates of N fertiliser does not continue to be related positively to crop yield, with over-fertilisation potentially leading to yield losses, associated with the hormetic effects of excess N on plant stomata^24^ and accumulation of nitrate in leafy vegetables^25^. Long-term, repeated application of N fertilisers has been linked to increases in soil organic carbon^26^, soil microbial biomass^27^ and bacterial diversity^28^ in agricultural soils, relative to unfertilised soil. However, application of urea or ammonium-based N fertiliser can lead to soil acidification over the long-term, predominantly caused by nitrification processes and base cation removal (via harvesting or leaching^29^). Thus, synthetic fertiliser addition associated with declining soil pH can become a positive reinforcing loop, acidifying soil and leading to declining yields unless soil pH is managed (Supplementary Fig. 3).

### Liming

Liming soil (adding calcium-based compounds) to replace harvested base cations^30^ and adjust soil pH for optimal crop growth conditions is practised in many countries. The pH adjustment of an acid soil toward the optimal pH for a crop has a positive effect on crop yield^30^. This is true in both the short- and long-term when farmers monitor their soil pH (Supplementary Fig. 3).

### Application of pesticides

Application of pesticides is considered an essential practice for producing high crop yields^31^ (see details in Supplementary Information and Supplementary Fig. 4). Pesticides are bioactive toxic substances used to control pests; they include herbicides, fungicides, insecticides, and nematicides. In the short-term, following pesticide application, crop yield increases^32^ through the combined effects of decreased competition between pests and the crop for nutrition and water, and through the reduced infestation of diseases and insects on the crops (Supplementary Fig. 4a).

Generally, pesticides are meant to have low environmental persistence and good biodegradation properties, but over the long term, they can accumulate in soils and harm non-target organisms^33^. The cumulative impact of improper or inappropriate use of pesticides leads to resistance in target and non-target insects, fungi, bacteria and weeds^34^, accumulation of pesticides (e.g, organochlorine, organophosphate) and their degradative products^32^ (metabolites; including heavy metals: e.g., Cu, Mn^35^) in soil which altogether entails negative effects on soil biota, soil processes and functions (Supplementary Fig. 4b). These long-term effects can have implications for exacerbating reinforcing feedback loops and crop yields.

### Plastic mulch films

Plastic mulches are used in agriculture to increase soil temperature, promote germination, protect crops, reduce soil water evaporation, support irrigation systems, reduce soil erosion and alter the spectral distribution and availability of light for the crop below^36^. After crop harvesting, the non-biodegradable plastic mulch film should be removed, to be incinerated or disposed of in landfill sites^37^, however this recovery is often neglected, and plastic residues can accumulate in agricultural soils^38^.

The short-term benefits of mulching with plastic films include accelerating germination, crop protection and shortening the growing season, allowing plants to be harvested sooner^39^ and having a positive relationship with crop yield (Supplementary Fig. 5a). In the long-term, repeated use and ploughing-in of plastic mulch films can contribute to reinforcing feedback loops associated with accumulation of plastic residues in soils, which affects soil conditions (including soil microbial composition, soil structure and physicochemical properties^40^) and has a toxic effect on soil biota^41^, thus undermining soil resilience and leading to yield reductions^38^ (further detailed in Supplementary Information).

### Irrigation in arid and semi-arid areas

Crop growth is often water-limited, and the use of irrigation systems, particularly in semi-arid and arid areas, can ensure crops receive an adequate water supply during the growing season. Currently, 22.5% of global cropland has irrigation systems in place^42^. Irrigation of crops can result in dramatic yield increases^43^ in both the short- and long-term (Supplementary Fig. 6). Consequently, irrigation is a significant management tool for farmers, which enhances crop yields from soils that might overwise produce low yields, with the global land area equipped for irrigation increasing by 11% between the years 2000 and 2015^44^.

However, where water resources are often limited, farmers can be compelled to use poor-quality water (e.g., brackish water) for irrigation. Indeed, much of the global expansion in irrigated lands has occurred in places that are water-stressed (where use exceeds supply^44^. Long-term, repeated irrigation with water containing elevated concentrations of ions (e.g., Na^+^ and Cl^-^) introduces salts to the agricultural system, increasing soil salinity over the longer term, with strong evaporative forces leaving the salts present in irrigation water on the soil surface^45^. Salinisation of soils significantly impacts soil resilience and the capacity to grow crops and a reinforcing feedback loop is observed when water limitation is managed with brackish water (Supplementary Fig. 6b).

### Flooding of paddy fields

Rice production under flooded conditions (also known as paddy soils) has been practised for millennia, and it includes many elements that make it very sustainable^46^. In the short-term, initial flooding of soils can result in a release of nutrients, meaning fertiliser inputs can be minimal^47^. The effects of flooding in the short-term, including adequate water supply and a reduction in pests and diseases, typically result in high and stable yields^46^ (Supplementary Fig. 7a). Over the long-term (Supplementary Fig. 7b) the uptake of intensive agricultural practices, such as the use of high yielding and fertiliser dependent rice varieties, mechanisation of farm practices and increased agrochemical inputs (fertilisers and pesticides), has enabled rice yields under flooded conditions to support 4.6 billion people as a staple food crop (2018 values^48^). Although the uptake of these technologies is associated with unsustainable externalities, the practice of flooding soil and paddy agriculture continues to deliver high rice yields and seemingly minimally affects the long-term resilience of these important agricultural soils^48^.

### Livestock grazing of intensively managed grassland

Typical characteristics of intensively managed grasslands are that they are sown with high-yielding plant varieties, grazed by livestock bred for high productivity, and managed at high stock densities. Grazing at optimal levels can increase primary productivity and potentially support a more diverse habitat. However, mis-managing grassland, e.g., by imposing stocking rates exceeding the carrying capacity^49^, can contribute to grassland degradation and desertification^50^. In the short-term, increasing stocking rates will increase meat/milk yields per hectare (see supplementary Information and Supplementary Fig. 8a). Herbage production can be increased through fertiliser inputs^51^, which will have a positive relationship with meat/milk yields (Supplementary Fig. 8a). Over the longer-term, large herds/flocks grazing the same amount of land will force the system into reliance on additional nutrient inputs as fertilisers, to replace the harvested nutrients and maintain grass yields (Supplementary Fig. 8b). Intensive grazing, particularly under wet conditions, can lead to soil compaction, which has a negative relationship with herbage production and, ultimately, the reduced ability to support livestock; i.e., decreased carrying capacity^52^ (Supplementary Fig. 8b). At the point where the intensively managed grassland can no longer support the growth of the herd/flock grazing it, additional feed is required to sustain production.

### Livestock grazing of rangeland

Rangelands include natural grassland, savanna, shrubland, many deserts, steppe, tundra, alpine communities and marshes^49^. Grazing animals are considered an integral part of many rangeland ecosystems. Whereas once rangelands supported large herds of grazing animals intrinsic to their landscape, the loss of these native animals makes traditional pastoral grazing a key component of habitat maintenance^53^. The sustainability of grazing rangelands is a topic of intense debate: some studies suggest large areas could carry greater numbers of livestock^54^; others state the current stocking rate of rangelands is approaching or has already exceeded any carrying capacity^55^, whilst others observe that the carrying capacity of rangelands varies spatially and temporally^56^.

In the short-term, grazing rangelands with livestock has a positive relationship with meat, milk and other animal product yields (Supplementary Fig. 9a). However, in the longer-term with inappropriate grazing practices this positive relationship is lost, with overstocking or overgrazing of rangelands considered a major factor in rangeland degradation^57^ (Supplementary Fig. 9b), leading to vegetation loss^58^, declining soil OM^59^ and soil erosion^58^. Continuous grazing of low-input grassland results in declining productivity over time, irrespective of the stocking rate^60^.

### Forest clearing and burning followed by fallow

Forest clearing and burning followed by a fallow period (or slash and burn or shifting cultivation) is driven by economic and demographic factors^61^. As the demand for agricultural land increases, deforestation via forest clearing and burning enables large areas of natural and semi-natural biomass to be cleared for the production of crops and livestock. The practice of forest clearing and burning followed by a fallow period, before repeated clearing, currently occurs in many tropical regions, due to food and nutritional security pressures^61^.

Initially, forest clearing and burning has a positive relationship with crop and timber yield, with enhanced nutrient and cation supply sourced from ash inputs into the soil supporting biomass growth^62^ (Supplementary Fig. 10a). In the longer term the soil is subject to many of the impacts that occur in other agricultural systems due to the lack of vegetative cover, including loss of OM, increased susceptibility to elevated erosion, declining fertility (unless fertilisers are used) and above- and below-ground biodiversity loss^63–65^ (Supplementary Fig. 10a). Agriculture on highly weathered tropical soil often involves little to no soil conservation measures and organic or inorganic nutrient replenishment is limited^66^. Subsequent fallow periods can ameliorate many of these impacts^64^, as well as other alternative practices (Table 1), but too short a fallow period results in declining crop and timber yields as a spiral of decline occurs.

### The complexity of multiple management options

The processes, feedbacks, impacts and systems diagrams associated with these stand-alone farm management practices (both in the short- and long-term) are presented in more detail in the Supplementary Information. However, agricultural land has diverse management systems, with farmers having multiple tools to address observed system performance. For example, in a tilled or forest clearing and burning system, both practices can lead to elevated soil erosion and deplete soil nutrients, which has a negative relationship with soil OM (Fig. 1b). We attempt to highlight the complexity of long-term conventional agricultural management practices in contrast to short-term effects for crops in Fig. 1 and for livestock systems in Fig. 2 and how these impacts affect our capacity to produce food from agricultural soils, combining the feedbacks identified from the literature for each individual management practice (Supplementary Figs. 1−10). In the short-term, the impacts of management activities on the yield potential of a soil are typically positive and have a positive relationship with yield (Figs. 1a and 2a), or the relationships between management activity and impact and between impact and yield are both negative which leads to an overall positive relationship between management impact and yield (e.g., spraying, flooding in Fig. 1a). Whereas, in the long-term, the repeated practice of some management activities can lead to soil impacts which negatively affect yield, such as OM decline, elevated soil erosion, salinisation, resistance, biodiversity decline, compaction, vegetation loss and accumulation/toxicity (Figs. 1b and 2b). Multiple management activities, including tillage, forest clearing and burning, irrigation, grazing of rangelands, and spraying, can be associated with soil impacts that negatively affect yield, which could be alleviated or exacerbated when used in combination with other management practices.

### Breaking the cycle

We have covered the impacts of conventional management of agricultural soils in the short- and long-term, however, there is evidence demonstrating that alternative farming practices can break or slow down the reinforcing cycle of impacts eroding soil resilience and the capacity to produce a yield from soil (Table 1). Thus, the negative impacts of conventional long-term management on soil resilience can be partially or fully negated through the application of alternative practices; however, most alternative practices come with trade-offs as well as benefits (Table 1).

**Table 1:** Alternative management practices that can aid in breaking the cycle of long-term inappropriate farming methods, with their benefits and trade-offs

Practice	Alternative practice	Benefits	Trade-offs	References
Tillage	Zero or no tillage	• Soil physical structure is maintained, including micro and macro porosity • Function of underground biota retained (e.g., mycorrhiza, nematodes) • Increase biodiversity • Tillage erosion reduced	• Increased weed pressure • Increased soil moisture can delay germination • Incorporation of organic fertilisers not always possible (although slurry injection is an option) and can lead to nitrogen losses via atmosphere • Benefits might vary over time (i.e., the seasonality effect on species and duration of the practice impact) • Temporary yield reduction • Soil texture and climate can influence crop productivity under zero tillage	Soil biota^16^ Yield reductions^17,85^ Biodiversity^86^ Tillage erosion^19^ Soil texture and climate effects^87^
	Reduced tillage (tillage depth, intensity, frequency or spatial coverage is reduced)	• Density and diversity of soil micro and meso-fauna increased • Allows soil incorporation of organic inputs	• Organic matter gains can be lost following a tillage event and will take time to build up again • Potential yield reduction	Soil biota^16^ Yield reductions^85^
	Use of organic fertilisers	• Can improve and valorise re-use of food, crop and livestock by-products • Organic fertilisers can increase soil organic matter content, soil water holding capacity • Reduces nutrient losses to environment associated with wasting by-products	• Uncertainty around and variability of nutritional composition, therefore challenging to optimise crop nutrition • Availability, transport and cost can be an issue • Potential source of contamination (e.g., heavy metals)	Benefits^88^ Trade-offs^89^ Contamination^90^
	Legume inclusion or rotation	• Reduces nitrogen fertiliser requirement • Legume yield may be greater under conditions that companion crops may decline under (e.g., ryegrass and clover leys at temperatures > 25°) • Legumes can have a legacy effect, increasing soil nitrogen supply for the next crop	• Biological nitrogen fixation capacity less in cooler climates	Increased sward yields and zero N fertiliser^51^
	Plant breeding (targeting nitrification inhibition or phosphorus and nitrogen-efficient plants)	• Increased nutrient efficiency reduces requirements for nitrogen and phosphorus fertilisers • Reduced emissions of nitrogen and phosphorus to the wider environment	• Availability of suitable varieties • Reduction in diversity/genetic erosion	Biological nitrification inhibition^91^ P utilisation efficiency^92^
	Apply nitrogen cycle inhibitors with the fertiliser	• Can improve crop nitrogen use efficiency • Reduces nitrogen emissions to air and water • Provides opportunity to reduce nitrogen application rate	• Effectiveness is dependent on local management and environmental conditions • Availability and cost can be an issue	Yield increase and effectiveness^93^ Yield increase and reduce fertilizer rate^94^
Pesticides	Plant breeding	Reduces need for pesticides and environmental impacts associated with pesticide use	Resistant varieties might be difficult to access for some growers	Benefits and trade-offs^95^
	Integrated pest management	• Can reduce the amount and variety of pesticides used • Cost savings made by reducing chemical inputs	Requires grower knowledge/understanding, including identification of different pests and understanding of their life cycle	Benefits and trade-offs^95^
Plastic mulching	Use truly biodegradable plastic with non-toxic compounds	• Provide micro-climate benefits without toxicity of plastic contamination	• Availability and price of materials	Benefits and trade-offs^96^
Irrigation	Use mulches	• Mulches can reduce evaporation from the soil surface	• Plastic mulches can lead to soil contamination	See SI5
	Increase soil organic matter	• Increases macro-aggregation in highly weathered soils	• Difficult to achieve without external organic material inputs in highly weathered soils	Benefits and trade-offs^97^
	Plant breeding for drought/salt-tolerant varieties	• Improves crop tolerance to drought/salt and reduces the need for other mitigative strategies	• Geographic and economic access to new varieties, • Legislative barriers for genetic editing, which vary by country and region	Benefits and trade-offs^98^
	Use of salt-tolerant rhizobacteria	• Potential to support plant growth and survival under saline conditions	• Plant response not consistent across varying global regions	Benefits and trade-offs^70^
Flooding of paddy fields	Alternate wetting and drying cycles	• Alternate wetting and drying can increase water use efficiency and reduce GHG emissions	• Possible yield penalty for alternate wetting and drying • Good alternate wetting and drying management is time-consuming and variable between fields • Alternate wetting and drying can lead to increased mineralisation of soil organic carbon • Good weed management is essential for alternate wetting and drying practices	Benefits and trade-offs^99^
	Straw management	• Improved straw management can reduce greenhouse gas emissions and maintain organic carbon input		Straw management^100^
	Plant breeding for nutrient use efficiency	• High NUE varieties are higher yielding with the same nutrient input	• High NUE varieties may have lower nutritional value	
Grazing intensive grassland	Diverse herbal leys, including N-fixing species	• Deep-rooting herbal leys improve soil porosity, aggregate stability, and topsoil and subsoil carbon storage; this in turn enhances water infiltration and nutrient cycling • Improved drought resistance	• Introduction of new herbal leys requires destruction of the previous crop via tillage and/or herbicides • Challenges with broadleaf weed control	Soil porosity^101^ Soil aggregates^102^ Soil carbon^103^ Drought resistance & trade-offs^104^
	Controlled grazing systems (mob or rotational grazing)	• Rotational grazing has potential for higher pasture carrying capacity • Rotational grazing can increase soil carbon content	• Mis-managed rotational grazing can result in compromised forage quality and therefore impair livestock performance • Requires more infrastructure and labour • Risk of poaching caused by high stocking densities on small paddocks in wet conditions	Benefits^105^ Trade-offs^104^
Grazing rangelands	Reduce stocking rates	• Adopting low and/or flexible stocking rates can help reduce land erodibility and limit accelerated erosion	• Reduction of grazing pressure or complete abandonment can lead to shrub encroachment	Benefits^106^ Trade-offs^107^
	Control grazing with herding	• Adaptive grazing strategies, which include controlled herding, can regenerate depleted soil and maintain plant integrity	• Implementing controlled grazing systems may involve upfront costs for infrastructure such as fencing and water systems	Benefits^108^ Trade-offs^106^
	Introduce pastures for nomadic grazing	• Yield of cultivated forage is substantially greater than that of the natural grassland	• Rapid degradation of re-established plant communities on bare-land due to poor management or poor soil structure and composition	Benefits and trade-offs^109^
Forest clearing and burning, followed by fallow	Increase the length of the fallow period	• Better biogeochemical starting conditions • Can support increases in the functional diversity of fallow area	• Greater land area needed to produce a similar yield	Benefits^64,110^
	Introduce high-yielding crop varieties	• Higher yields • Improved economic and social outcomes	• Cost and difficulty of accessing remote places • Introduction of commodity crops can increase deforestation via a rebound effect. • Modern varieties may require greater nutrient inputs, which may lead to clearing of primary forest.	Higher yields^111^ Rebound effect^112^ High yields, economic and social outcomes, and nutrient demands^113^
	Introduce fertilizers	• Higher yields	• Cost and difficulty of accessing remote places	Nutrient demands^113^
	Agroforestry	• Multiple products produced (e.g., timber, fuel, fibre, feed, pharmaceuticals, alley/surrounding ground crops) • Co-benefits (shade, pollinators, habitat for game, soil nutrient fixation) • Improved economic and social outcomes	• Finding good rotation • Competition with crop • Unwanted impacts of tree species on local hydrology	Benefits^65,114^

### Significance of conventional agricultural management practices for soil resilience

Soils take a long time to form, with it taking about 1000 years to generate a depth of 25 mm of soil, although it can be faster in some regions^67^. Therefore, we consider soil loss via elevated erosion to be the most important impact of feedback loops driven by agricultural management practices^68^. This is because in the timespans considered here, elevated soil loss is effectively permanent, and without soils, crops cannot be grown. Therefore, any feedback loop associated with elevated soil erosion should be prioritised (Figs. 1 and 2). This means inappropriate tillage, rangeland grazing, and forest clearing and burning are the management systems that pose the greatest extant and pervasive threat to soil resilience and food production. In these cases, the removal of vegetation and exposure of bare soil to water and wind erosion processes typically cause elevated soil erosion. This is exacerbated by the loss of soil OM, which promotes the degradation of soil structure and aggregate stability, and under tillage, the development of plough-pans, which prevent infiltration of effective rainfall. Weakened soil aggregates in combination with reduced infiltration and consequential enhanced surface runoff means elevated soil erosion is highly likely. It is predicted that future cropland expansion will be greatest in Sub-Saharan Africa, South America and Southeast Asia, meaning the least developed economies will be open to the greatest threats to soil resilience^69^. The extent of overgrazing and associated reductions in the carrying capacity of grazed lands are also key factors in the high positioning of overgrazing of rangelands^56^.

We consider salinisation caused by inappropriate irrigation the next most important feedback loop discussed here. This is primarily because of (i) its extent, affecting 20% of irrigated land^70^; (ii) its difficulty to reverse, and; (iii) the uncertainty around its impact on soil biota^71^. Contamination of soils caused by the introduction of toxic components via pesticide spraying and plastic mulching follows salinisation in order of importance, respectively, due to their eventual toxicity to soil and the difficulties in reversing it (see Supplementary Information). Compaction of soils and the inputs required to sustain soils that are under intensive grazing are the next most important impacts, due to the difficulty in reversing soil compaction and the ongoing dependency on continued inputs of agrochemicals (Supplementary Fig. 8 and Supplementary Information). Soil acidification associated with N fertiliser inputs follows, although this impact is not challenging to reverse in countries where lime or other resources that neutralise soil pH are available. However, this is not the case for many countries and for many farmers. Liming and flooding share the status of the least consequential feedback loops presented here, due to the unlikelihood of liming being carried out beyond an optimal soil pH and the long-term productivity of flooded soils.

### Feedback loops and the potential for passing critical thresholds in agricultural soils

Lastly, we highlight the potential danger of unexpected and unintended consequences. Human activities have the potential to push ecosystems past critical thresholds at which even a small perturbation can qualitatively alter the state or development of a system – termed ‘tipping points’^3,72^. Given the importance of agricultural systems, abrupt changes in soil resilience and the consequent effect on food and feed yields resulting from tipping points in agricultural soils could be speculated to cause near ‘end-of-world’ scenarios^73^. Whilst not all the reinforcing feedback loops identified above will lead to tipping points, prudent risk management requires consideration of these bad-to-worst-case scenarios^74^. Although not considered here, the potential cumulative impacts on soil systems when faced with the inappropriate application of the conventional management practices described herein, in combination with the impacts of climate change, have the potential to cause system collapses much sooner and more pervasively than individual drivers acting alone^3^.

Systems theory suggests that for various tipping points, early warning signals prior to system collapse may be detectable. For soils, early warnings might include non-responsive soils with crop yields unaffected by nutrient inputs^75^. A great deal of research has focused on identifying early warning metrics linked to critical slowing down theory^1^ and alternative approaches to identifying resilience loss in real systems prior to tipping points through structural metrics^76,77^. Examples of such early warnings within the context of soil are few. However, changes in soil moisture autocorrelation have been identified as an early warning signal for drought-induced food crises, with a lead time of up to three to six months for every case^78^. Ongoing research into soil functionality and soil resilience driven by the soil microbiome may provide further tools to understand declines in soil resilience^9,79,80^ and to identify soil systems approaching critical thresholds. Systems may be able to evade tipping points via spatial re-organisation^81^. Future studies should investigate whether spatial re-organisation can provide signals of decline of agricultural soils resilience, although early warning signals may be less likely to be observed when driven by multiple, fast drivers and extreme events^3,82^. Further work is also needed to understand the potential for human intervention to prevent critical transitions in soils. Extension services have not been reviewed here, with this study focussing on soil processes. However, the impact of the training and support farmers receive from extension services can be substantial for improving soil management^83^, not only for mitigating the impacts of agricultural practices on soil resilience, but also for closing yield gaps and reducing the effects of agriculture on wider externalities^84^.

## Conclusions

Resilient soils are essential for food and feed security, ecological diversity, and for their role in the global carbon cycle. Yet, inappropriate conventional farming practices over the long term can threaten the resilience of agricultural soils and may undermine food and feed security. Herein, we have examined the scientific literature to support the conceptualisation of short- and long-term impacts of multiple agricultural management practices and presented them as feedback loops, applying a “systems thinking” approach to the resilience of agricultural soils. We have summarised these system loops into overarching figures of multiple management practices and highlighted the complexity of managing soils to support their resilience and associated food production. We find that in the short term, many agricultural practices can enhance food and feed yields, which leads to the continuation of the practice. However, long-term repetition of some agricultural practices can have impacts that threaten soil resilience and reduce yields. We suggest that practices which cause soil loss via elevated erosion pose the greatest risk to soil resilience, highlighting inappropriate tillage, forest clearing and burning, and overgrazing of rangelands as examples of primary threats to global soil resilience. Lastly, we suggest that concepts of resilience, critical thresholds, risk management and systems theory are of fundamental importance for the management of resilient agricultural soils.

## Supplementary information

Supplementary information

## Data Availability

No datasets were generated or analysed during the current study.
