# Supplementary material for: Agricultural practices can threaten soil resilience through changing feedback loops
Source: NPJ Sustain Agric. 2025 Oct 1;3(1):56. doi: 10.1038/s44264-025-00098-6 (PMC12488477; doi:10.1038/s44264-025-00098-6)
Supplement: Supplementary file 1 — Supplementary information [file 44264_2025_98_MOESM1_ESM.pdf]

Supplementary Information file for: "Agricultural practices can threaten soil resilience through changing feedback loops"

Authors: Alison M. Carswell*[1], Simon Willcock[1,2], Martin S.A. Blackwell[1], Hari Ram Upadhayay[1], Paul Harris[1], Graham McAuliffe[1], Andrew L. Neal[1], M. Jordana Rivero[1], Laura M. Cardenas[1], Stephan M. Haefele[3], Andrew P. Whitmore[4], John A. Dearing[5], Fusuo Zhang[6], Mark Farrell[7,8], Marijn Bauters[9], Pascal Boeckx[10], Yuri Jacques A.B. da Silva[11], Kwame Agyei Frimpong[12], Adrian Collins[1]

Affiliations:

1. Net Zero & Resilient Farming, Rothamsted Research, North Wyke, Okehampton EX20 2SB, UK; alison.carswell@rothamsted.ac.uk, simon.willcock@rothamsted.ac.uk, martin.blackwell@rothamsted.ac.uk, hari.upadhayay@rothamsted.ac.uk, gmcauliffe@harper-adams.ac.uk, paul.harris@rothamsted.ac.uk, andy.neal@rothamsted.ac.uk, jordana.rivero.visiting@rothamsted.ac.uk, laura.cardenas@rothamsted.ac.uk, adrian.collins@rothamsted.ac.uk
2. School of Environmental and Natural Sciences, Bangor University, Bangor, Gwynedd, LL57 2DG, United Kingdom;
3. Sustainable Soils and Crops, Rothamsted Research, Harpenden AL5 2JQ, United Kingdom; stephan.haefele@rothamsted.ac.uk
4. Net Zero & Resilient Farming, Rothamsted Research, West Common, Harpenden, AL5 2JQ, United Kingdom; andy.whitmore@rothamsted.ac.uk
5. School of Geography and Environmental Science, University of Southampton, Southampton, SO17 1BJ, United Kingdom; j.dearing@soton.ac.uk
6. State Key Laboratory of Nutrient Use and Management, College of Resources and Environmental Sciences, National Academy of Agriculture Green Development, National Observation and Research Station of Agriculture Green Development (Quzhou, Hebei), China Agricultural University, Beijing, China; zhangfs@cau.edu.cn
7. CSIRO Agriculture & Food, Kaurna Country, PO Box 200, Glenside, Adelaide, SA 5064, Australia; mark.farrell@csiro.au
8. UWA School of Agriculture & Environment, The University of Western Australia, Whadjuk Noongar Country, 35 Stirling Highway, Perth, WA 6000, Australia;
9. Department of environment, Ghent University, Ghent, Belgium; marijn.bauters@ugent.be
10. Department of green Chemistry and Technology, Ghent University, Ghent, Belgium; pascal.boeckx@ugent.be
11. Agricultural Engineering Department, Federal Rural University of Pernambuco, Dom Manuel de Medeiros street, s/n - Dois Irmãos, 52171-900 Recife, Brazil; yuri.silva@ufrpe.br
12. Department of Soil Science, School of Agriculture,College of Agriculture and Natural Sciences, University of Cape Coast, Cape Coast, Ghana; kfrimpong@ucc.edu.gh

*Corresponding author: alison.carswell@rothamsted.ac.uk

System diagrams approach

The system diagrams presented below were generated after reviewing the scientific literature for the impacts of agricultural management practices on soils and food and feed yields. The labelled boxes indicate relevant factors, which have the potential to be influenced by each management practice. Farmer actions (i.e., leading to the management intervention, often in response to yield or profit fluctuations) are shown as dotted arrows, while any knock-on effects of these actions are shown as solid arrows and demonstrate the influence of one factor on another (e.g., a '+' showing a positive relationship between two factors, and a '-' indicating a negative relationship). In each case, the starting point is taken to be a soil under natural or semi-natural vegetation, which is converted to agricultural production.

Supplementary Information 1: Tillage

Tillage is practiced in both arable and pasture/ley-based agriculture, having originated 10-13 millennia ago[1]. Its intensity can vary ranging from gentle scraping of the soil surface to a shallow depth, to deep harrowing (disturbing the soil to depths of around 20 cm using tines or discs) or ploughing (turning of the soil to depths of around 20 cm). The latter are considered 'conventional tillage'. Today it is typically performed either annually or on a scheduled basis prior to sowing a new crop or sward. It serves three main purposes: i) to eliminate competition from weeds meaning herbicide use is reduced; ii) to prepare a good seedbed (e.g., to increase the area of contact between the seed and the soil) into which the next crop can be sown, iii) it promotes soil nutrient availability for crops through the mineralisation of soil organic matter (OM), and; iv) it can alleviate compaction. However, the mineralisation and associated loss of soil OM has consequences for: i) soil structure making it, more vulnerable to compaction and saturation during rainfall events due to poor infiltration, more vulnerable to low oxygen tensions and so liable to emissions of nitrous oxide and methane as a result of anaerobic microbial respiration, as well as erosion via water or wind [2]; ii) soil water holding capacity, making it vulnerable to drought[3]; iii) soil nutrients; as they become rapidly available from mineralised OM they are at risk of being mined; iv) vulnerability to erosion, due to exposure of bare soil to wind and rain coupled with loss of structure and strength due to the decrease in soil OM and lack of plant roots binding the soil (as well as providing protection from rainfall kinetic energy). In the short-term, yield is supported due to conventional tillage, and potentially yield gains can occur as nutrients become readily available when soil OM is mineralised, causing the practice to continue (Supplementary Figure 1a). There are additional negative consequences resulting from tillage which can affect crop yields, including increased nutrient losses, associated either with soil erosion, leaching and flushes of greenhouse gases, as well as mortality of beneficial invertebrates (e.g., earthworms) and the disruption of fungal hyphal networks, following conventional tillage events. However, these are more likely to become consequential after repeated tillage rather than in the short-term.

Eventually the effects of tillage on soil OM will result in a decline in crop yield due to increases in the negative effects listed above (Supplementary Figure 1b). The degree of decline in soil OM content at which a significant impact occurs, or even complete crop failure takes place, varies depending on soil type and climate. Over time, declining yields are likely to prompt land managers to apply greater rates of fertilisers (organic or synthetic/mined). Added in combination, these can result in maintenance of crop yields[4-6], and increase soil OM levels (although effects of inorganic fertilisers vary in different soils), but cannot restore all the original characteristics of the soil. Long-term tillage may continue to affect earthworm diversity and abundance[7], leaching of nutrients, and reduce or eliminate infiltration of water past the tillage layer if an impermeable "plough pan" is formed, promoting elevated surface runoff and erosion. Development of impermeable layers caused by tillage are typically addressed by strategic deep tillage, however the impact of tillage on biological and biochemical processes which support soil aggregate stability[8] is not easily repaired. These impacts of long-term repeated tillage can result in a positive reinforcing feedback loop (Supplementary Figure 1b), leading farmers to become reliant of externally sourced inputs (see also

Supplementary Figure 2) and heavy farm machinery, or to seek alternative approaches to manage weeds and provide a good seedbed.

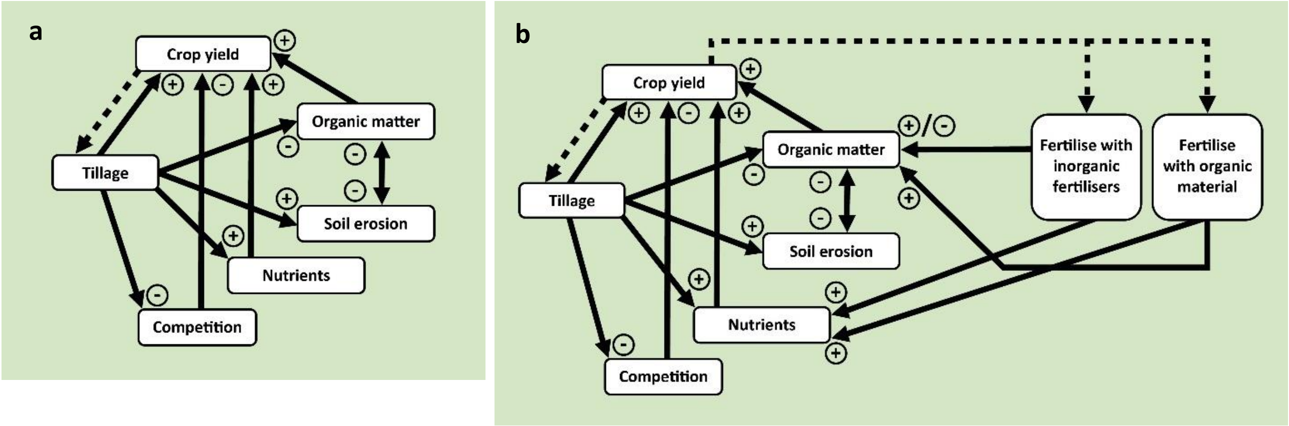

Supplementary Figure 1 – Systems diagrams illustrating the short-term (a) and long-term (a) impacts of ploughing on agricultural soil. *As a result of the declining yields, land managers react by adding fertilisers and manures. The labelled boxes indicate relevant factors, which have the potential to be impacted by each management practice. The actions of the farmer (i.e., leading to the management intervention, often in response to yields or profits) is shown with a dashed arrow, while the knock-on effects of these actions are arrived at by solid black arrows. The positive or negative symbols indicate the direction of the relationship between the two factors (e.g., a '+' showing a positive relationship between two factors, and a '-' indicating a negative relationship).*

Supplementary Information 2: Application of Fertilisers

Fertilisers can be divided into two categories; there are fertilisers derived from organic residues (including livestock manures, composts, sewage sludge and other recycled organic by-products) which may in themselves originate from agricultural production. These recycled fertilisers have supported agricultural outputs since civilisations settled and have potential to contribute to sustainable agricultural systems[9,10]. Recycled fertilisers are composed of nutrients that if not returned to agricultural land would be wasted, exacerbating the already disrupted global biochemical flows[11]. Where recycled fertilisers are well-managed in agricultural systems, they provide macro- and micro-nutrients to crops and are an important source of OM. However, when over-applied without consideration for soil nutrient status and crop nutrient requirements, soil can become saturated, leading to elevated nutrient losses via water or the atmosphere. The second fertiliser category are external inputs to the agricultural system, sourced via industrial synthesis or mineral extraction. Our work focuses on nitrogen (N) fertiliser as the dominant fertiliser applied to soils globally. These fertilisers have revolutionised agriculture, with reactive N produced via the Haber-Bosch process providing food for approximately half the global population[12]. As above, we focus on the short- and long-term effects – this time of synthetic N fertiliser applications. In the short-term, applications of synthetic N fertiliser are associated with increased yields (Supplementary Figure 2a). These beneficial outcomes lead to the continuation of N fertilisation as a regular practice. Negative effects of N fertiliser applications (both in the short- and long-term) can occur when system N use efficiency is low and surplus N is high[13,14]. In addition, there are examples of negative yield responses to N applications in dryland systems, where water scarcity leads to crops 'haying off' after good growth at anthesis[15,16]. Although, where water availability is sufficient, application of N fertilisers is typically linked with a positive yield response in the short-term.

Supplementary Figure 2b shows that repeated use of synthetic N fertilisers can have a varied relationship with yield, as described in the main article. Changes in soil pH can have negative implications for crop N use efficiency (and other nutrient use efficiencies), with declining soil pH towards acidic or increasing soil pH towards alkaline both having a negative relationship with crop yield, whereas neutral soils will be optimal for nutrient use efficiency (Supplementary Figure 2b). Thus, soil acidification caused by inappropriate synthetic fertiliser addition can become a positive reinforcing loop (see also Supplementary Figure 3).

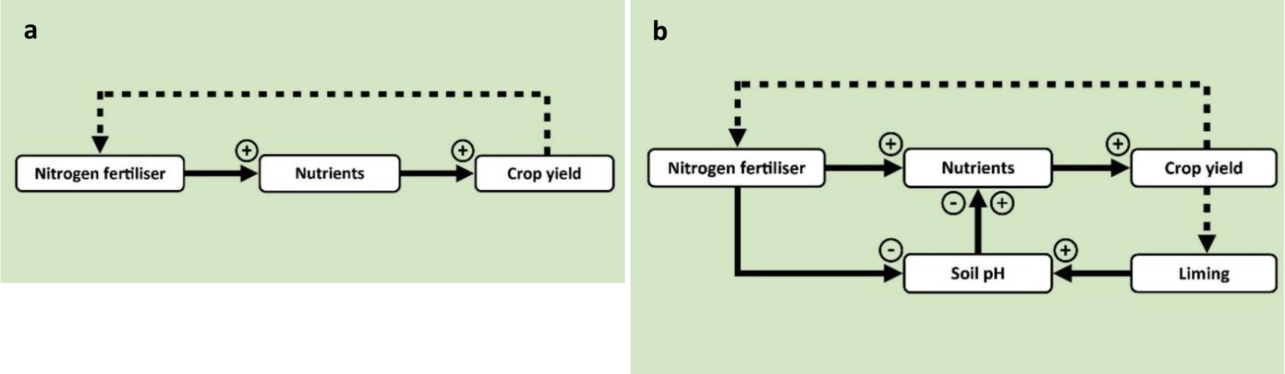

Supplementary Figure 2 – Systems diagrams illustrating the short-term (A) and long-term (B) impacts of applying synthetic nitrogen fertiliser to agricultural soil. *The format of the diagram follows Supplementary Figure 1.*

Supplementary information 3: Liming

Soil acidification is a common problem of intensive agricultural systems, caused by: (i) removal of base cations by harvested crops; (ii) acidifying fertilisers (including sulphur and urea or ammonium-based N); (iii) acidifying precipitation and deposition; (iv) leaching of base cations, and; (v) mineralisation of soil OM[17-19]. We do not consider liming of soil in the short or long-term to have any detrimental impacts on soil resilience nor crop yields, unless in an unusual case where lime is over-applied and soil becomes alkaline (Supplementary Figure 3). However, this is deemed very unlikely due to the implied economics of liming through product, transport, and labour costs.

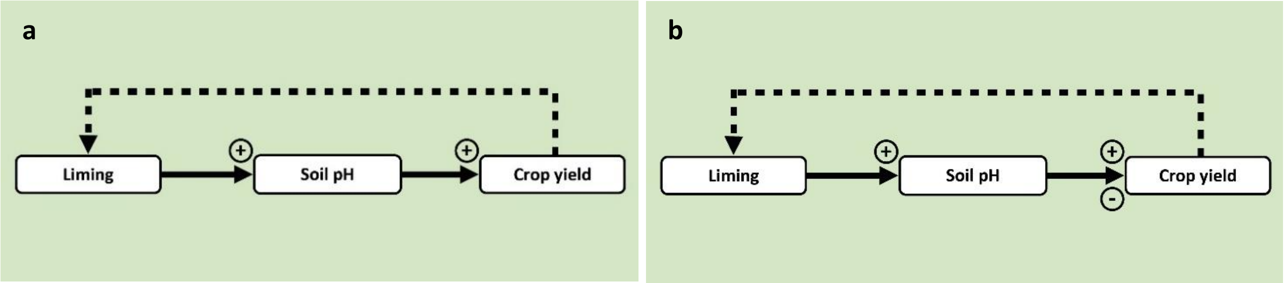

Supplementary Figure S3 – Systems diagrams illustrating the short-term (a) and long-term (b) impacts of liming on agricultural soil. *The format of the diagram follows Supplementary Figure 1.*

Supplementary Information 4: Application of pesticides

The short and long-term consequences of pesticide application on soil and crop yield is shown in Supplementary Figure 4 and are described in the main article. This agricultural practice is considered critical for obtaining high levels of crop productivity (OECD and FAO 2012). For example, yield gains in winter wheat due to pesticide application ranges from 0.3 t ha^{-1} to 2.3 t ha^{-1} [20,21].

However, pesticide use is associated with increased resistance of target species, and yield losses can occur because of this. In England annual wheat yield loss due to herbicide-resistant black grass is ~0.8 million tonnes[22]. Other impacts of inappropriate pesticide use on soils include destroying microbial structural proteins and suppression of dehydrogenase, phosphatase and nitrogenase activities[23]. Soil microbial biomass, particularly arbuscular mycorrhizal fungi, is reduced by pesticide residues in soil[24]. Deleterious effects of herbicide applications upon earthworms[25] reduces the mixing of organic litter within the soil and

soil nutrient cycling and availability, all of which may lead to reductions in crop productivity[26]. Schneider et al.[27] remark that estimates of yield losses attributed to stopping pesticide use often overlook the potential for positive ecological feedbacks to affect and potentially protect against yield losses.

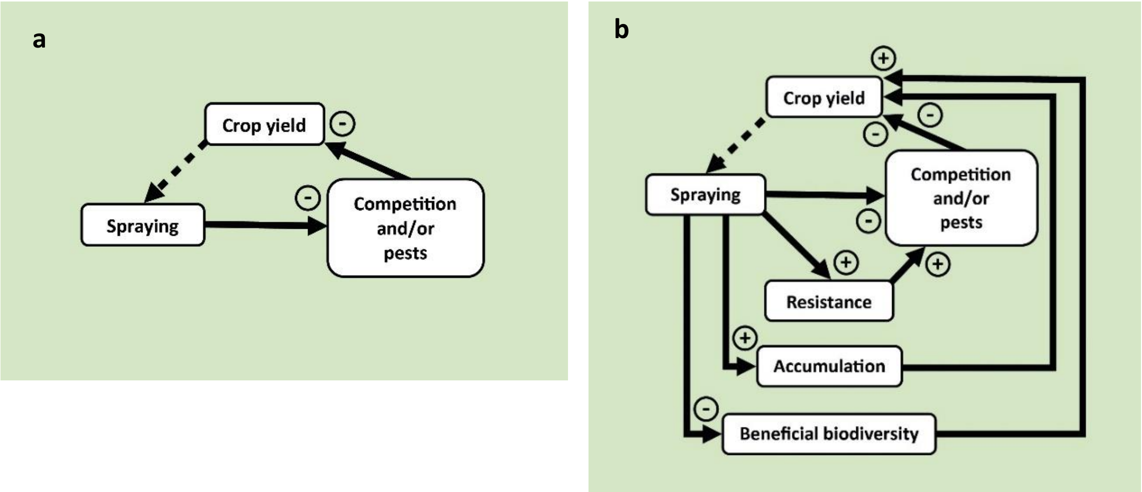

Supplementary Figure 4 ***– Systems diagrams illustrating the short-term (a) and long-term (b) impacts of spraying pesticides on agricultural soil.*** *The format of the diagram follows Supplementary Figure 1.*

Supplementary Information 5: Plastic mulch films

Plastic mulches have been used in global agriculture since the 1960s, initially in colder regions to increase soil temperature, promote germination and protect crops. They are now used globally to control soil moisture and light availability[28]. The FAO[29] estimates that 7.5 Mt of plastic films are used in terrestrial agriculture annually. These immediate benefits are indicated by the positive relationship between plastic mulching and soil environmental conditions in the short term; e.g., temperature and moisture (Supplementary Figure 5a). Plastic mulches can also help reduce competition from weeds and lower herbicide and pesticide requirements in the short term, although this is largely dealt with by the fact that the practice is nearly always accompanied by ploughing (see above; Supplementary Figure 1). However, as well as protecting crops, plastic mulch films offer protection to, and favourable conditions for, pests such as molluscs and pathogenic fungi[30]. Thus, it is common practise to apply pesticides and fungicides, along with fertilisers (collectively inputs; Supplementary Figure 5), at the time of mulch film installation. Compared to conventional crop production, plastic mulch films generally require more labour and/or specialist equipment to facilitate their installation, but ultimately this results in increased profit in the short-term due to the benefits described (Supplementary Figure 5a). After crop harvesting, non-biodegradable films are removed[31], whereas biodegradable films are ploughed back into the soil as part of conventional soil management[31]. However, full recovery of plastic mulch films is often neglected, leading to the accumulation of plastic residues in agricultural soils[32].

Long-term repeated use and ploughing-in of plastic mulch films can have negative consequences, especially if the mulch films used are not truly biodegradable in the short-term or non-biodegradable films are not recovered (Supplementary Figure 5b[32]). Many films are designed to simply break into smaller particles, through to micro- and nano-plastics and there is increasing evidence that plastics can become incorporated into organisms and ultimately the food chain[33]. Further, accumulation of plastic residues can negatively affect soil conditions and lead to yield reductions, as estimated by Zhang et al.[32] with a 6 – 10% reduction in cotton yield in a field containing 317 kg plastic residue ha^{-1}, which offset the yield gains made from the adoption of intensive agricultural practices. Contamination of soils with plastic residues, alone and in combination with other contaminants, has a toxic effect on soil biota (Supplementary Figure 5b[33]) and has a negative impact on soil conditions. Additionally, increases in pests (e.g., mycotoxigenic fungi[30]) under plastic mulch films can increase pesticide use, further reducing profit and resulting in increased soil contamination (see above; Supplementary Figure 4).

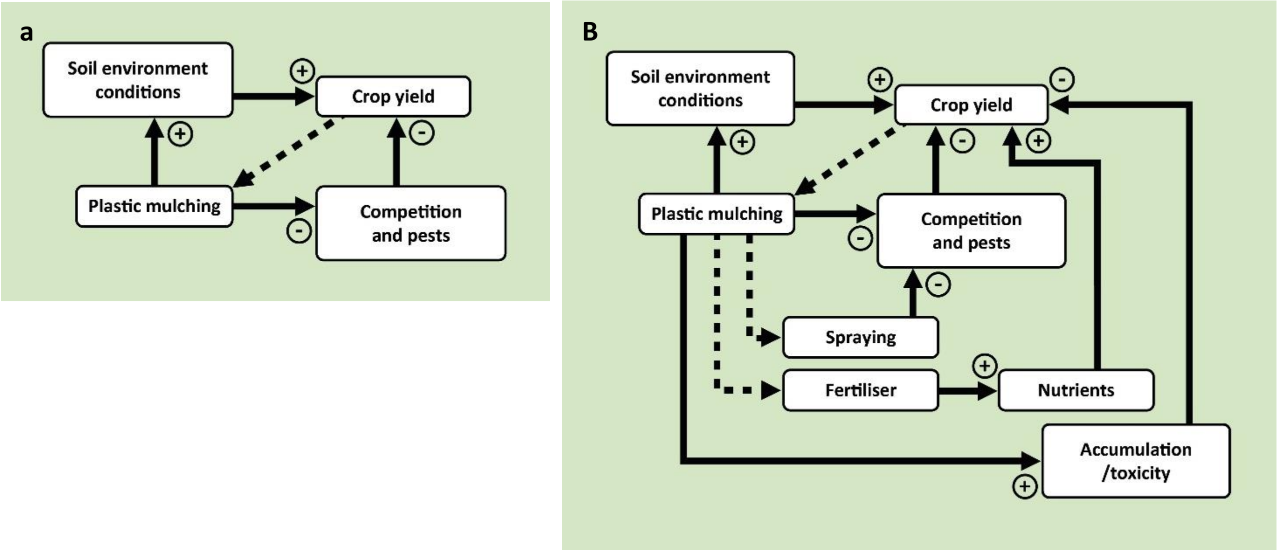

Supplementary Figure 5 – Systems diagrams illustrating the short-term (a) and long-term (b) impacts of plastic mulch films on agricultural soil. *The format of the diagram follows Supplementary Figure 1.*

Supplementary Information 6: Irrigation in arid and semi-arid areas

Irrigation systems are used by farmers to ensure crops receive adequate water supply during the growing season. In the short term, irrigation of crops can result in dramatic yield increases (Fig S6A). In the USA, Kukal and Irmak[34] reported that irrigation limited yield gaps were equivalent to 4.12, 1.47 and 1.00 t ha^{-1} for maize, winter wheat and soybean, respectively, whereas in Northeast China, Liu et al.[35] suggested that suboptimal water supply contributed to between 6 and 48% of the maize yield gaps in Northeast China. In arid and semi-arid areas with limited water resources, farmers may use poor-quality water (e.g., brackish water) for irrigation. Irrigation with brackish water can cause soil salinity (Supplementary Figure 6b[36]). Moderate soil salinity (8 – 10 dS m^{-1}) results in yield losses of 55%, 28%, and 15% in maize, wheat and cotton, respectively. High salinity (18 dS m^{-1}) results in 55% yield losses in cotton[37]. Some crops are more sensitive to salinity than others, with vegetable crops deemed more sensitive to salt stress than cereal crops, especially during early growth stages (germination, establishment[38]). Globally, the economic impact of saline soils is estimated to range from 12.7 – 27.3 billion US$ per year[39,40] and estimates suggest that worldwide, approximately 1 billion ha of soils are salt-affected, by both natural and anthropogenic processes[41].

Surface irrigation increases salinity in deeper soil layers, while in furrow irrigation systems, salts accumulate in ridges of soil between the furrows[42]. In the long term, inappropriate irrigation (water quality, quantity, timing, methods) as well as other farming practices (e.g., application of high salt index fertilizer, ploughing down or tilling deeper) further exacerbate salt accumulation in soils, significantly reducing crop productivity. Moreover, increasing soil salinity is pronounced in low-lying coastal areas associated with sea-level rise due to climate change and salinisation due to saltwater intrusion into depleted aquifers[43]. Flushing and leaching of salt (the dissolving and translocation of soluble salts below 45–60 cm soil depth) with irrigation water is an important farming practice to increase crop productivity by managing root zone salinity in regions with high evapotranspiration and low rainfall. However, the irrigation required to do this can result in secondary salinisation and exacerbate yield losses[44].

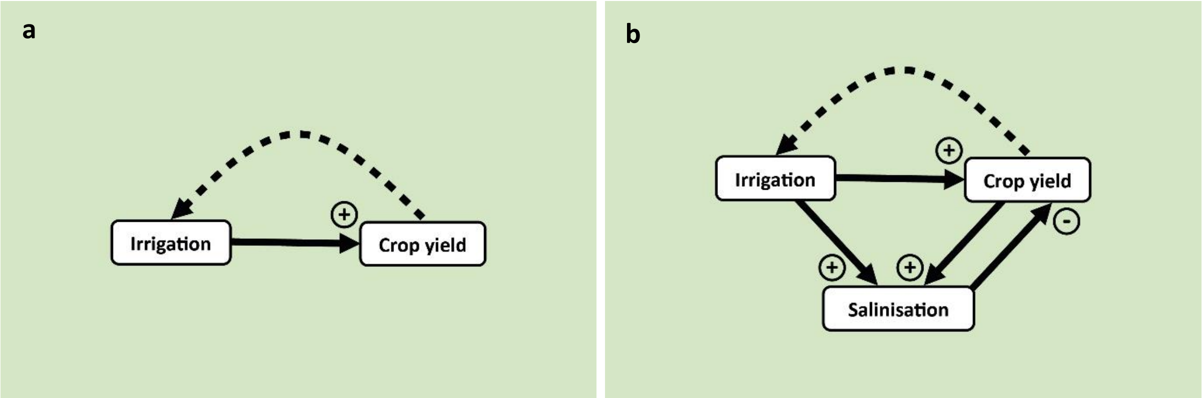

Supplementary Figure 6 – Systems diagrams illustrating the short-term (a) and long-term (b) impacts of irrigation on agricultural soil. *The format of the diagram follows Supplementary Figure 1.*

Supplementary Information 7: Flooding

In the short-term, flooding of soils results in a release of nutrients and a reduction in pests and diseases[45], which generally results in high and stable yields (Supplementary Figure 7a). Over the long-term, repeated flooding of soils has little impact on soil resilience and use of flooding along with the uptake of other intensive agricultural practices, like fertiliser addition, has further allowed rice yields to increase and it remains a staple food for billions of people (Supplementary Figure 7b[46]).

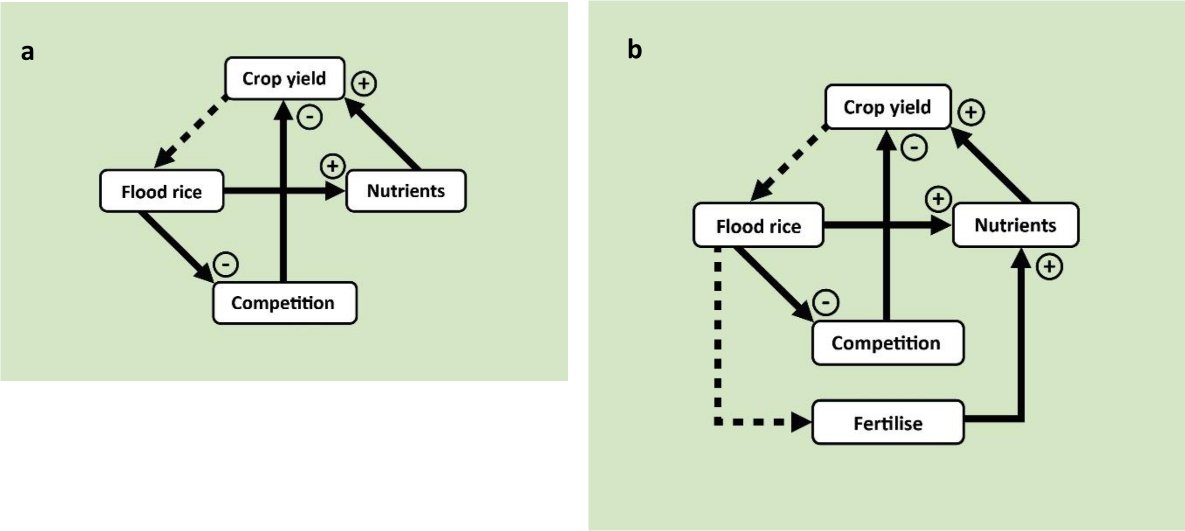

Supplementary Figure 7 – Systems diagrams illustrating the short-term (a) and long-term (b) impacts of flooding rice on agricultural soil. *The format of the diagram follows Supplementary Figure 1.*

Supplementary Information 8: Livestock grazing of intensively managed grassland

Grazing land accounts for 70% of the world's agricultural land (in 2022[47]) and can be divided into three main categories: natural, semi-natural and intensively managed grasslands. We focus on intensively managed grasslands. Herbage production and consequently meat/milk yields can increase in the short-term, with good sward management and replacement of harvested nutrients and cations with agrochemical inputs (Supplementary Figure 8a). Over the long-term, repeated or more intensive grazing will lead to the need to replace harvested nutrients to maintain herbage production (Supplementary Figure 8b) and potentially compact soils which limits herbage production through the reduced access to soil water and nutrients[48]. If herbage production declines to a point where it cannot support the local herd/flock, then external feed is sourced to finish the livestock to optimal slaughter weights.

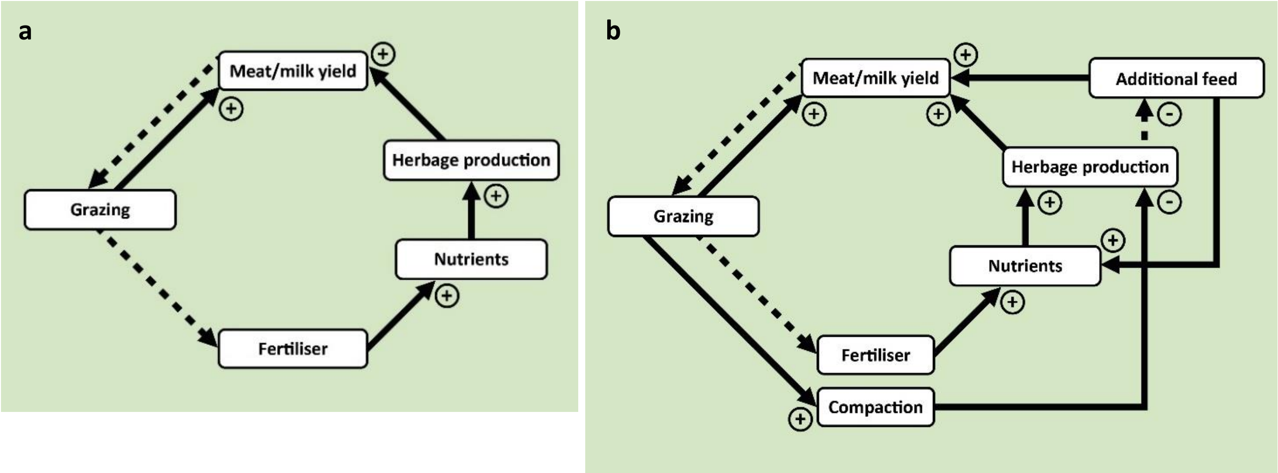

Supplementary Figure 8 – Systems diagrams illustrating the short-term (a) and long-term (b) impacts of livestock grazing on improved grasslands. *The format of the diagram follows Supplementary Figure 1.*

Supplementary Information 9: Livestock grazing of rangeland

Rangelands contain indigenous plants and are natural ecosystems used to produce livestock[49]. Stocking of rangelands is carried out by varying land management stakeholders, such as single households or organisations versus management by multiple households or organisations[50]. In the short-term, allowing livestock to graze rangelands has a positive relationship with meat/milk yields (Supplementary Figure 9a). However, with inappropriate stocking, in the long-term, this trend can be reversed (Supplementary Figure 9b) with increased grazing leading to greater vegetation loss which reduces meat/milk yields and has negative implications for soil erosion and soil OM[51-53]. Rangeland degradation is recognised globally as a threat to these important ecosystems. Overgrazing has a key role in this degradation, but climate change is also recognised as an important, and at times, driving factor[54,55].

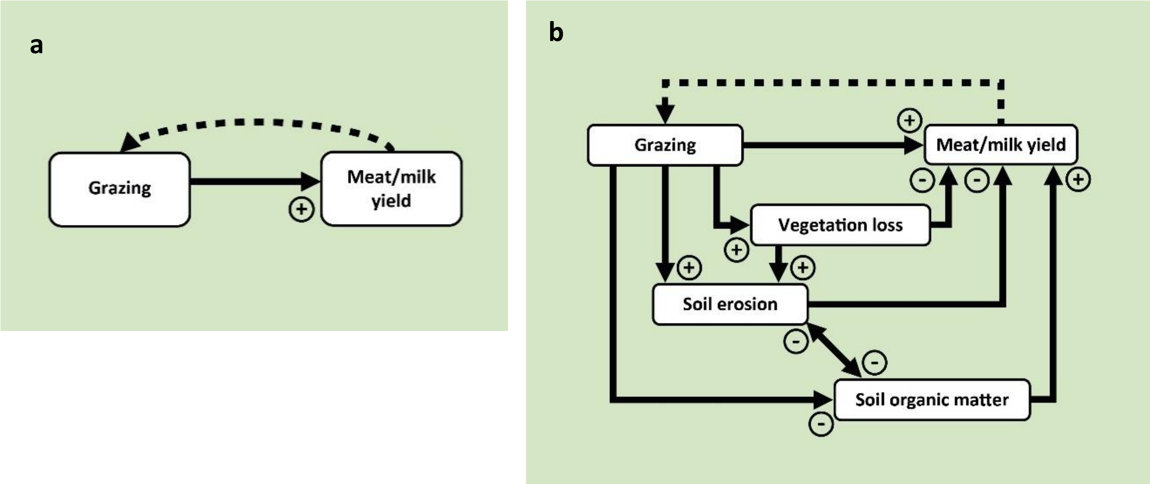

Supplementary Figure 9 – Systems diagrams illustrating the short-term (a) and long-term (b) impacts of livestock grazing on rangelands. *The format of the diagram follows Supplementary Figure 1.*

Supplementary Information 10: Forest clearing and burning followed by fallow

The practice of forest clearing and burning followed by a fallow period in the short-term has a positive relationship with crop or timber yield, which is largely driven by an immediate increase in soil nutrition (Supplementary Figure 10a). Forest clearing and burning (or slash and burn or shifting cultivation) is driven by economic and demographic factors[56]. However, in the long-term or over repeated cycles the process of forest clearing and burning is associated with soil erosion and reduced soil OM, reduced soil nutrients and reduced biodiversity (Supplementary Figure 10b). Changing the length of the fallow period is an additional practice, which can help manage soil nutrient content (see Table 1 main article; Supplementary Figure 10b).

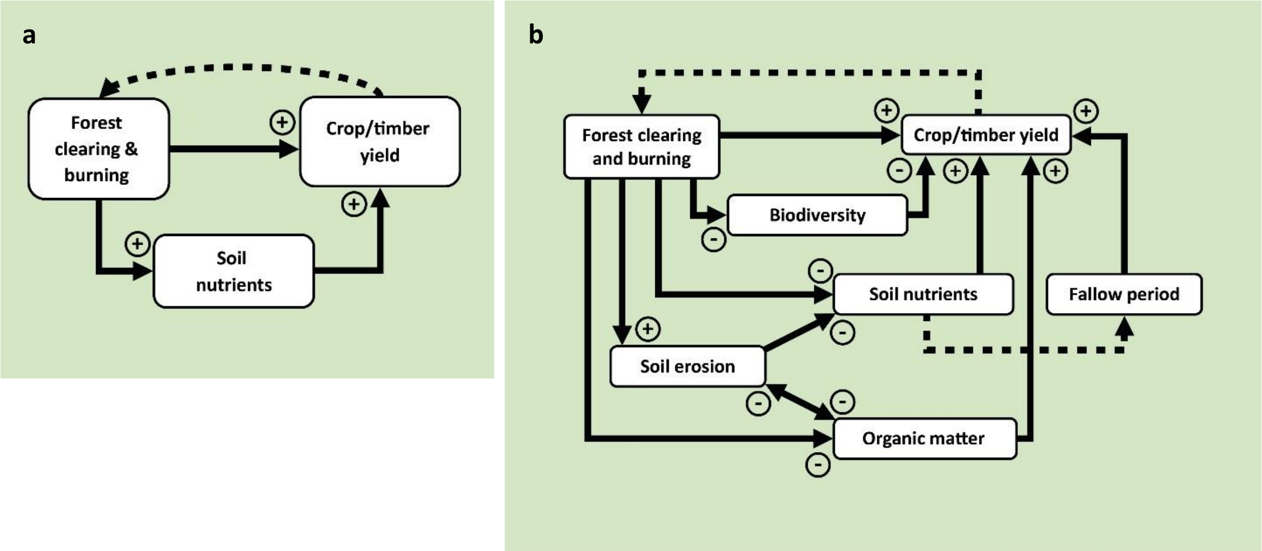

Supplementary Figure 10 – Systems diagrams illustrating the short-term (a) and long-term (b) impacts of slash and burn/shifting cultivation. *The format of the diagram follows Supplementary Figure 1.*

Supporting information References:

1 Lal, R., Reicosky, D. C. & Hanson, J. D. Evolution of the plow over 10,000 years and the rationale for no-till farming. *Soil and Tillage Research* **93**, 1–12 (2007). https://doi.org/https://doi.org/10.1016/j.still.2006.11.004

2 Montgomery, D. R. Soil erosion and agricultural sustainability. *Proceedings of the National Academy of Sciences* **104**, 13268–13272 (2007). https://doi.org/doi:10.1073/pnas.0611508104

3 Haddaway, N. R. *et al.* How does tillage intensity affect soil organic carbon? A systematic review. *Environmental Evidence* **6**, 30 (2017). https://doi.org/10.1186/s13750-017-0108-9

4 Wei, W. *et al.* Effects of combined application of organic amendments and fertilizers on crop yield and soil organic matter: An integrated analysis of long-term experiments. *Agriculture, Ecosystems & Environment* **225**, 86–92 (2016). https://doi.org/https://doi.org/10.1016/j.agee.2016.04.004

5 Research, R. (ed Rothamsted Research Electronic Rothamsted Archive) (2017a).

6 Research, R. (ed Rothamsted Research lectronic Rothamsted Archive) (2017b).

7 Ernst, G. & Emmerling, C. Impact of five different tillage systems on soil organic carbon content and the density, biomass, and community composition of earthworms after a ten year period. *European Journal of Soil Biology* **45**, 247–251 (2009). https://doi.org/https://doi.org/10.1016/j.ejsobi.2009.02.002

8 Chellappa, J., Sagar, K. L., Sekaran, U., Kumar, S. & Sharma, P. Soil organic carbon, aggregate stability and biochemical activity under tilled and no-tilled agroecosystems. *Journal of Agriculture and Food Research* **4**, 100139 (2021). https://doi.org/https://doi.org/10.1016/j.jafr.2021.100139

9 Billen, G. *et al.* Reshaping the European agro-food system and closing its nitrogen cycle: The potential of combining dietary change, agroecology, and circularity. *One Earth* **4**, 839–850 (2021). https://doi.org/10.1016/j.oneear.2021.05.008

10 Sutton, R., Howarth, R. W., Mason, K. E., Brownlie, W. & Cordovil, C. Nitrogen opportunities for Agriculture, Food & Environment. (2022).

11 Steffen, W., Broadgate, W., Deutsch, L., Gaffney, O. & Ludwig, C. The trajectory of the Anthropocene: The Great Acceleration. *The Anthropocene Review* **2**, 81–98 (2015). https://doi.org/10.1177/2053019614564785

12 Erisman, J. W., Sutton, M. A., Galloway, J., Klimont, Z. & Winiwarter, W. How a century of ammonia synthesis changed the world. *Nature Geoscience* **1**, 636–639 (2008). https://doi.org/10.1038/ngeo325

13 Lassaletta, L. *et al.* Nitrogen use in the global food system: past trends and future trajectories of agronomic performance, pollution, trade, and dietary demand. *Environmental Research Letters* **11**, 095007 (2016). https://doi.org/10.1088/1748-9326/11/9/095007

14 Guo, M. C. *et al.* How China's nitrogen footprint of food has changed from 1961 to 2010. *Environmental Research Letters* **12** (2017). https://doi.org/10.1088/1748-9326/aa81d9

15 van Herwaarden, A. F., Angus, J. F., Richards, R. A. & Farquhar, G. D. 'Haying-off', the negative grain yield response of dryland wheat to nitrogen fertiliser II.Carbohydrate and protein dynamics. *Australian Journal of Agricultural Research* **49**, 1083–1094 (1998). https://doi.org/https://doi.org/10.1071/A97040

16 Borrell, A. K., Christopher, J. T., Kelly, A., Collins, B. & Chenu, K. Balancing pre- and post-anthesis growth to maximise water-limited yield in cereals. *Field Crops Research* **296**, 108919 (2023). https://doi.org/https://doi.org/10.1016/j.fcr.2023.108919

17 van Breeman, N., Mulder, J. & Driscoll, C. T. Acidification and alkalinization of soils. *Plant and Soil* **75**, 283–308 (1983).

18 Goulding, K. W. T. Soil acidification and the importance of liming agricultural soils with particular reference to the United Kingdom. *Soil Use and Management* **32**, 390–399 (2016). https://doi.org/https://doi.org/10.1111/sum.12270

19 Xu, D., Carswell, A., Zhu, Q., Zhang, F. & de Vries, W. Modelling long-term impacts of fertilization and liming on soil acidification at Rothamsted experimental station. *Science of The Total Environment* **713**, 136249 (2020). https://doi.org/https://doi.org/10.1016/j.scitotenv.2019.136249

20 Hossard, L. *et al.* Effects of halving pesticide use on wheat production. *Scientific Reports* **4**, 4405 (2014). https://doi.org/10.1038/srep04405

21 Wiik, L. Yield and disease control in winter wheat in southern Sweden during 1977–2005. *Crop Protection* **28**, 82–89 (2009). https://doi.org/https://doi.org/10.1016/j.cropro.2008.09.002

22 Varah, A. *et al.* The costs of human-induced evolution in an agricultural system. *Nature Sustainability* **3**, 63–71 (2020). https://doi.org/10.1038/s41893-019-0450-8

23 Tejada, M., García, C., Hernández, T. & Gómez, I. Response of Soil Microbial Activity and Biodiversity in Soils Polluted with Different Concentrations of Cypermethrin Insecticide. *Archives of Environmental Contamination and Toxicology* **69**, 8–19 (2015). https://doi.org/10.1007/s00244-014-0124-5

24 Riedo, J. *et al.* Widespread Occurrence of Pesticides in Organically Managed Agricultural Soils—the Ghost of a Conventional Agricultural Past? *Environ. Sci. Technol.* **55**, 2919–2928 (2021). https://doi.org/10.1021/acs.est.0c06405

25 Gaupp-Berghausen, M., Hofer, M., Rewald, B. & Zaller, J. G. Glyphosate-based herbicides reduce the activity and reproduction of earthworms and lead to increased soil nutrient concentrations. *Scientific Reports* **5**, 12886 (2015). https://doi.org/10.1038/srep12886

26 van Groenigen, J. W. *et al.* Earthworms increase plant production: a meta-analysis. *Scientific Reports* **4**, 6365 (2014). https://doi.org/10.1038/srep06365

27 Schneider, K., Barreiro-Hurle, J. & Rodriguez-Cerezo, E. Pesticide reduction amidst food and feed security concerns in Europe. *Nature Food* **4**, 746–750 (2023). https://doi.org/10.1038/s43016-023-00834-6

28 Kasirajan, S. & Ngouajio, M. Polyethylene and biodegradable mulches for agricultural applications: a review. *Agronomy for Sustainable Development* **32**, 501–529 (2012). https://doi.org/10.1007/s13593-011-0068-3

29 FAO. Assesment of agricultural plastics and their sustainability. A call for action. (Food and Agricultural Organization of the United Nations, Rome, 2021).

30 Steinmetz, Z. *et al.* Plastic mulching in agriculture. Trading short-term agronomic benefits for long-term soil degradation? *Science of The Total Environment* **550**, 690–705 (2016). https://doi.org/https://doi.org/10.1016/j.scitotenv.2016.01.153

31 Kitamoto, H. *et al.* Accelerated degradation of plastic products via yeast enzyme treatment. *Scientific Reports* **13**, 2386 (2023). https://doi.org/10.1038/s41598-023-29414-1

32 Zhang, D. *et al.* Plastic pollution in croplands threatens long-term food security. *Global Change Biology* **26**, 3356–3367 (2020). https://doi.org/https://doi.org/10.1111/gcb.15043

33 Azeem, I. *et al.* Uptake and Accumulation of Nano/Microplastics in Plants: A Critical Review. *Nanomaterials (Basel)* **11** (2021). https://doi.org/10.3390/nano11112935

34 Kukal, M. S. & Irmak, S. Irrigation-limited yield gaps: trends and variability in the United States post-1950. *Environmental Research Communications* **1**, 061005 (2019). https://doi.org/10.1088/2515-7620/ab2aee

35 Liu, Z. J. *et al.* Climate zones determine where substantial increases of maize yields can be attained in Northeast China. *Climatic Change* **149**, 473–487 (2018). https://doi.org/10.1007/s10584-018-2243-x

36 Pulido-Bosch, A. *et al.* Impacts of agricultural irrigation on groundwater salinity. *Environmental Earth Sciences* **77**, 197 (2018). https://doi.org/10.1007/s12665-018-7386-6

37 Satir, O. & Berberoglu, S. Crop yield prediction under soil salinity using satellite derived vegetation indices. *Field Crops Research* **192**, 134–143 (2016). https://doi.org/https://doi.org/10.1016/j.fcr.2016.04.028

38 Li, R., Shi, F., Fukuda, K. & Yang, Y. Effects of salt and alkali stresses on germination, growth, photosynthesis and ion accumulation in alfalfa (Medicago sativa L.). *Soil Science and Plant Nutrition* **56**, 725–733 (2010). https://doi.org/10.1111/j.1747-0765.2010.00506.x

39 Qadir, M. *et al.* Economics of salt-induced land degradation and restoration. *Natural Resources Forum* **38**, 282–295 (2014). https://doi.org/https://doi.org/10.1111/1477-8947.12054

40 Wang, F., Yang, S., Wei, Y., Shi, Q. & Ding, J. Characterizing soil salinity at multiple depth using electromagnetic induction and remote sensing data with random forests: A case study in Tarim River Basin of southern Xinjiang, China. *Science of The Total Environment* **754**, 142030 (2021). https://doi.org/https://doi.org/10.1016/j.scitotenv.2020.142030

41 ITPS, F. a. Status of the World's Soil Resources (SWSR) - Main Report. (Food and Agriculture Organization of the United Nations and Intergoernmental Technical Panel on Soils, Rome, Italy, 2015).
42 Zaman, S., Shahid, S. A. & Heng, L. in *Guideline for salinity assessment, mitigation and adaptation using nuclear and related techniques* Ch. 4, 91–111 (Springer nature, 2018).
43 Daliakopoulos, I. N. *et al.* The threat of soil salinity: A European scale review. *Science of The Total Environment* **573**, 727–739 (2016). https://doi.org/https://doi.org/10.1016/j.scitotenv.2016.08.177
44 Arora, N. K. *et al.* Environmental sustainability: challenges and viable solutions. *Environmental Sustainability* **1**, 309–340 (2018). https://doi.org/10.1007/s42398-018-00038-w
45 Peng, S., Tang, Q. & Zou, Y. Current Status and Challenges of Rice Production in China. *Plant Production Science* **12**, 3–8 (2009). https://doi.org/10.1626/pps.12.3
46 Yanai, J. *et al.* Long-term changes in paddy soil fertility in tropical Asia after 50 years of the Green Revolution. *European Journal of Soil Science* **73**, e13193 (2022). https://doi.org/https://doi.org/10.1111/ejss.13193
47 FAO. (ed FAO) (Rome, Italy, 2024).
48 Drewry, J. J., Cameron, K. C. & Buchan, G. D. Pasture yield and soil physical property responses to soil compaction from treading and grazinga review. *Soil Research* **46**, 237–256 (2008). https://doi.org/https://doi.org/10.1071/SR07125
49 Allen, V. G. *et al.* An international terminology for grazing lands and grazing animals. *Grass and Forage Science* **66**, 2–28 (2011). https://doi.org/https://doi.org/10.1111/j.1365-2494.2010.00780.x
50 Cao, J., Yeh, E. T., Holden, N. M., Yang, Y. & Du, G. The effects of enclosures and land-use contracts on rangeland degradation on the Qinghai–Tibetan plateau. *Journal of Arid Environments* **97**, 3–8 (2013). https://doi.org/https://doi.org/10.1016/j.jaridenv.2013.05.002
51 Zhao, H. L., Zhao, X. Y., Zhou, R. L., Zhang, T. H. & Drake, S. Desertification processes due to heavy grazing in sandy rangeland, Inner Mongolia. *Journal of Arid Environments* **62**, 309–319 (2005). https://doi.org/https://doi.org/10.1016/j.jaridenv.2004.11.009
52 Fenetahun, Y. *et al.* Impact of Grazing Intensity on Soil Properties in Teltele Rangeland, Ethiopia. *Frontiers in Environmental Science* **9** (2021). https://doi.org/10.3389/fenvs.2021.664104
53 Grinnell, N. A., Komainda, M., Tonn, B., Hamidi, D. & Isselstein, J. Long-term effects of extensive grazing on pasture productivity. *Animal Production Science* **63**, 1236–1247 (2023).
54 Eriksen, S. E. H. & Watson, H. K. The dynamic context of southern African savannas: investigating emerging threats and opportunities to sustainability. *Environmental Science & Policy* **12**, 5–22 (2009). https://doi.org/https://doi.org/10.1016/j.envsci.2008.10.009
55 Miao, L., Sun, Z., Ren, Y., Schierhorn, F. & Müller, D. Grassland greening on the Mongolian Plateau despite higher grazing intensity. *Land Degradation & Development* **32**, 792–802 (2021). https://doi.org/https://doi.org/10.1002/ldr.3767
56 van Vliet, N. *et al.* Trends, drivers and impacts of changes in swidden cultivation in tropical forest-agriculture frontiers: A global assessment. *Global Environmental Change* **22**, 418–429 (2012). https://doi.org/https://doi.org/10.1016/j.gloenvcha.2011.10.009
